# Test-retest reliability and construct validity of the ENERGY-parent questionnaire on parenting practices, energy balance-related behaviours and their potential behavioural determinants: the ENERGY-project

**DOI:** 10.1186/1756-0500-5-434

**Published:** 2012-08-13

**Authors:** Amika S Singh, Mai JM Chinapaw, Léonie Uijtdewilligen, Froydis N Vik, Wendy van Lippevelde, Juan M Fernández-Alvira, Sarolta Stomfai, Yannis Manios, Maria van der Sluijs, Caroline Terwee, Johannes Brug

**Affiliations:** 1 EMGO Institute for Health and Care Research, Department of Public and Occupational Health, VU University medical center, Amsterdam, the Netherlands; 2 Department of Public Health, Sport and Nutrition, University of Agder, Kristiansand, Norway; 3 Department of Public Health, Ghent University, Ghent, Belgium; 4 GENUD (Growth, Exercise, NUtrition and Development) research group, Escuela Universitaria de Ciencias de la Salud, Universidad de Zaragoza, Zaragoza, Spain; 5 Department of Paediatrics, University of Pécs, Pécs, Hungary; 6 Department of Nutrition and Dietetics, Harokopio University, Athens, Greece; 7 ResCon, Amsterdam, the Netherlands; 8 EMGO Institute for Health and Care Research, Department of Epidemiology and Biostatistics, VU University medical center, Amsterdam, the Netherlands; 9 EMGO Institute for Health and Care Research, VU University medical center, Amsterdam, the Netherlands

**Keywords:** Parental questionnaire, Proxy report, Psychometric, Physical activity, Obesity, Prevention, Validation, Reliability

## Abstract

**Background:**

Insight in parental energy balance-related behaviours, their determinants and parenting practices are important to inform childhood obesity prevention. Therefore, reliable and valid tools to measure these variables in large-scale population research are needed. The objective of the current study was to examine the test-retest reliability and construct validity of the parent questionnaire used in the ENERGY-project, assessing parental energy balance-related behaviours, their determinants, and parenting practices among parents of 10–12 year old children.

**Findings:**

We collected data among parents (n = 316 in the test-retest reliability study; n = 109 in the construct validity study) of 10–12 year-old children in six European countries, i.e. Belgium, Greece, Hungary, the Netherlands, Norway, and Spain. Test-retest reliability was assessed using the intra-class correlation coefficient (ICC) and percentage agreement comparing scores from two measurements, administered one week apart. To assess construct validity, the agreement between questionnaire responses and a subsequent interview was assessed using ICC and percentage agreement.

All but one item showed good to excellent test-retest reliability as indicated by ICCs > .60 or percentage agreement ≥ 75%. Construct validity appeared to be good to excellent for 92 out of 121 items, as indicated by ICCs > .60 or percentage agreement ≥ 75%. From the other 29 items, construct validity was moderate for 24 and poor for 5 items.

**Conclusions:**

The reliability and construct validity of the items of the ENERGY-parent questionnaire on multiple energy balance-related behaviours, their potential determinants, and parenting practices appears to be good. Based on the results of the validity study, we strongly recommend adapting parts of the ENERGY-parent questionnaire if used in future research.

## Background

There is a need for more carefully developed interventions to curb the obesity epidemic in youth. Prevention of unnecessary weight gain should target energy intake and energy expenditure behaviours, also referred to as energy balance-related behaviours (EBRBs), i.e. physical activity, sedentary, and dietary behaviours [1]. Development of effective interventions requires insight into which specific EBRBs are most relevant and into the correlates and determinants of these behaviours.

Parental and home-environmental factors are crucial determinants of children’s EBRBs and related weight status [1].

The ENERGY-cross sectional study [2] surveyed more than 7000 children, their parents, and schools in seven countries representing different regions of Europe using questionnaires aiming children, one of their parents, school staff as well as school environment observations. We wanted to assess a range of parental and home environmental factors with the parent questionnaire. However, no established valid and reliable measures that could be administrated in large population studies across Europe were available.

A similar study was conducted to test the child questionnaire [3]. We found that the ENERGY-child questionnaire has good test-retest reliability and moderate to good construct validity for the large majority of items. Therefore, a parent questionnaire was developed and a test-retest reliability and construct validity study was conducted. In the current paper, the methods and results of the test-retest reliability and construct validity study of the parent questionnaire are presented and discussed.

## Findings

### Methods

#### ENERGY-parent questionnaire

The ENERGY-parent questionnaire assessed behaviours and potential determinants regarding soft drinks and fruit juices, breakfast, dieting, physical activity, screen viewing, and sleeping of the child. The potential determinants included automaticity of behavioural choices, home availability, parenting practices and (economic) environmental factors.

Most of these concepts were assessed by one or two items. All correlates, except questions about family rules, were assessed on 5-point scales, with response categories ranging from ‘I fully agree’ to ‘I fully disagree’ or ‘Always’ to ‘Never’. Details on the development of the questionnaire, the pre-testing, and translation procedures are described elsewhere [4]. The ENERGY-parent questionnaire is available in English and all languages in which the questionnaire was administered via the ENERGY-website: http://www.projectenergy.eu.

#### Study population: recruitment and data collection

In six countries the test-retest reliability and construct validity study was conducted (Belgium, Greece, Hungary, the Netherlands, Norway, Spain) according to the standardized protocol described hereafter and in the Additional file 1. Due to deviations from the study protocol, the data from a seventh country (Slovenia) were excluded from the current study.

We recruited parents of children aged 10–12 years old. The recruitment and data collection took place from March until July 2010. Parents were recruited via 1) the schools of children participating in the children test-retest/construct validity study [3] or 2) other schools that the research team had personal contacts with.

In countries where ethical approval was necessary for such non-intervention studies this was obtained from the relevant ethical committee.

##### - Test-retest reliability study

When the parents agreed to participate in the test-retest reliability study, we emphasized the importance of the fact that the questionnaires must be completed on a weekday, and once more by the same person, exactly one week later. We sent the questionnaires to the home address of the parents 3–4 days apart; a stamped addressed envelope to return the questionnaire was included with both.

##### - Construct validity study

Parents who participated in the validity study were asked to fill in the questionnaire and were subsequently interviewed by a researcher or research assistant. The interview was performed using a standard question route. The interviews were recorded and transcribed. Based on the transcribed interview, a second researcher or research assistant (i.e. other than the one doing the interview) filled in a second identical parent questionnaire without knowledge of the answers to the first questionnaire of the parent.

#### Statistical analyses

We calculated means and standard deviations (participant characteristics) and medians, 25^th^, and 75^th^ percentiles values (EBRBs).

For both test-retest reliability and construct validity we assessed agreement at the individual item level. The agreement of categorical, continuous, and dichotomous items was analysed with a two-way random effects single measure intraclass correlation coefficient (ICC 2.1). ICCs were classified as ‘excellent’ (≥ .81), ‘good’ (.61 – .80), ‘moderate’ (.41 - .60), and ‘poor’ (≤ .40) [5-8].

Since the calculation of ICCs depends on the existence of the variability in answering categories, we also calculated percentage agreement, with criteria established as ‘excellent’ (90% - 100%), ‘good’ (75% - 89%), ‘moderate’ (60%-74%), or ‘poor’ (< 60%). If ICC values were lower than .40/.60/.80 but the percentage agreement was equal to or higher than 60%/75%/90%, we reported the percentage agreement [9].

All statistical tests were performed using SPSS version 18.0 (SPSS Inc., Chicago, IL).

## Results

### General

The characteristics of the participants from both the test-retest reliability and construct validity study are shown in Table 1.

**Table 1:** Descriptive statistics of the parents that participated in the test-retest reliability and construct validity study

	**Belgium**	**Greece**	**Hungary**	**Netherlands**	**Norway**	**Spain**
TEST-RETEST RELIABILITY STUDY*						
N in reliability study	70	56	50	52	39	49
Age, years (mean (sd))	40.3 (4.1)	40.7 (6.0)	38.4 (5.0)	43.7 (5.6)	40.8 (4.5)	39.7 (4.4)
% male study participants	20	27	10	23	23	20
BMI, kg/m^2^ (mean (sd))	24.2 (3.6)	25.0 (3.6)	24.8 (4.3)	25.2 (4.0)	25.0 (3.5)	25.4 (4.2)
Marital status (%)						
Single	4	0	10	2	0	2
Married/Living together with partner without being married/having a partner but not living together	89	96	80	81	84	88
Divorced/Separated	6	4	10	18	17	8
Other	0	0	0	0	0	2
Level of education (%)						
Less than 7 years	0	2	0	0	0	0
7-9 years	3	9	2	8	3	12
10-11 years	1	13	34	4	5	6
12-13 years	23	36	26	15	31	16
14 or more years	71	41	38	73	62	65
CONSTRUCT VALIDITY STUDY**						
N in validity study	20	20	20	20	20	9
Age, years (mean (sd))	37.4 (4.5)	42.8 (5.3)	38.7 (4.3)	44.0 (7.6)	42.6 (5.1)	41.8 (5.2)
% male study participants	15	30	10	25	55	0
BMI, kg/m^2^ (mean (sd))	24.5 (2.7)	24.5 (2.6)	24.5 (5.4)	24.5 (3.5)	no data	22.1 (2.0)
Marital status (%)						
Single	10	0	5	15	0	0
Married/Living together with partner without being married/having a partner but not living together	80	100	75	75	100	89
Divorced/Separated	5	0	20	10	0	11
Other	0	0	0	0	0	0
Level of education (%)						
less than 7 years	0	0	0	0	0	0
7-9 years	0	30	10	5	0	0
10-11 years	0	35	15	5	0	0
12-13 years	15	25	25	25	0	11
14 or more years	80	10	50	65	100	89

** Missing data test-retest reliability study: level of education: n = 1 (Belgium: n = 1).

* Missing data construct validity study: marital status: n = 5 (Belgium: n = 5); level of education: n = 5 (Belgium: n = 5).

The number of participants in the test-retest reliability study ranged from 39 (Norway) to 70 (Belgium). Completion of the questionnaire took 25–50 minutes.

In the construct validity study all countries included 20 parents, except for Spain (9 parents).

The majority of the interviews were carried out face-to-face, except for Belgium and Greece, where almost all interviews were carried out via telephone. The interviews took about 60 minutes.

Table 2 presents descriptive statistics of the parental energy balance-related behaviours.

**Table 2:** Energy balance-related behaviours of parents participating in the test-retest reliability and construct validity study

	**All**	**Belgium**	**Greece**	**Hungary**	**Netherlands**	**Norway**	**Spain**
**TEST-RETEST RELIABILITY STUDY**	n = 316	n = 70	n = 56	n = 50	n = 52	n = 39	n = 49
Soft drink consumption (ml/day)	77 (21 – 266)	71 (24 – 249)	36 (18 – 154)	202 (36 – 463)	59 (0 – 306)	71 (18 – 214)	83 (41 – 283)
Fruit juice consumption (ml/day)	107 (36 – 250)	71 (31 – 201)	107 (37 – 384)	83 (46 – 268)	107 (18 – 549)	107 (31 – 214)	124 (36 – 458)
Breakfast consumption (days/week)	7.0 (6. 0 – 7.0)	7.0 (7.0 – 7.0)	5.0 (1.0 – 7.0)	7.0 (5.0 – 7.0)	7.0 (6.3 – 7.0)	7.0 (7.0 – 7.0)	7.0 (7.0 – 7.0)
Cycling to/from work (min/day)	0.0 (0.0 – 0.0)	0.0 (0.0 – 0.0)	0.0 (0.0 – 0.0)	0.0 (0.0 – 0.0)	0.0 (0.0 – 5.7)	0.0 (0.0 – 0.0)	0.0 (0.0 – 0.0)
Walking to/from work (min/day)	0.0 (0.0 – 0.0)	0.0 (0.0 – 0.0)	0.0 (0.0 – 0.0)	0.0 (0.0 – 5.2)	0.0 (0.0 – 0.0)	0.0 (0.0 – 0.0)	0.0 (0.0 – 4.8)
Sports (min/day)	25.7 (2.1 – 38.6)	25.7 (8.6 – 36.4)	15.0 (0.0 – 28.9)	17.1 (8.6 – 25.7)	25.7 (1.1 – 42.9)	34.3 (25.7 – 51.4)	25.7 (2.1 – 38.6)
Watching TV (min/day)	100.7 (60.0 – 154.3)	81.4 (60.0 – 120.0)	96.4 (48.2 – 148.9)	115.7 (68.6 – 180.0)	98.6 (47.1 – 141.4)	162.9 (128.6 – 188.6)	85.7 (64.3 – 127.5)
Computer usage (min/day)	30.0 (8.6 – 60.0)	30.0 (17.1 – 38.6)	30.0 (0.0 – 55.7)	21.4 (0.0 – 53.6)	34.3 (21.4 – 60.0)	30.0 (8.6 – 68.6)	30.0 (8.6 – 68.6)
**CONSTRUCT VALIDITY STUDY**	n = 109	n = 20	n = 20	n = 20	n = 20	n = 20	n = 9
Soft drink consumption (ml/day)	71 (36 – 214)	107 (36 – 240)	59 (36 – 174)	119 (41 – 717)	18 (0 – 214)	107 (36 – 154)	94 (40 – 222)
Fruit juice consumption (ml/day)	107 (18 – 250)	74 (18 – 250)	214 (107 – 250)	83 (19 – 506)	36 (4 – 107)	107 (22 – 241)	179 (40 – 250)
Breakfast consumption (days/week)	7.0 (7.0 – 7.0)	7.0 (7.0 -7.0)	7.0 (5.3 – 7.0)	4.5 (1.3 – 7.0)	7.0 (7.0 – 7.0)	7.0 (7.0 – 7.0)	7.0 (7.0 – 7.0)
Cycling to/from work (min/day)	0.0 (0.0 – 4.7)	0.0 (0.0 – 0.0)	0.0 (0.0 – 0.0)	0.0 (0.0 – 6.8)	1.3 (0.0 – 6.0)	7.9 (0.0 – 10.4)	0.0 (0.0 – 0.0)
Walking to/from work (min/day)	0.0 (0.0 – 0.5)	0.0 (0.0 – 0.0)	0.0 (0.0 – 3.0)	0.0 (0.0 – 3.0)	0.0 (0.0 – 0.0)	0.0 (0.0 – 0.5)	2.1 (0.0 – 8.0)
Sports (min/day)	25.7 (17.1 – 42.9)	25.7 (17.1 – 51.4)	42.9 (21.4 – 60.0)	12.9 (8.6 – 27.9)	17.1 (8.6 – 40.7)	34.3 (25.7 – 42.9)	25.7 (17.1 – 51.4)
Watching TV (min/day)	96.4 (60.0 – 137.1)	111.4 (60.0 – 150.0)	132.9 (67.5 – 157.5)	81.4 (42.9 – 132.9)	120.0 (70.7 – 143.6)	83.6 (47.1 – 98.6)	85.7 (55.7 – 113.6)
Computer usage (min/day)	30.0 (8.6 – 60.0)	30.0 (8.6 – 38.6)	70.7 (21.4 – 160.7)	15.0 (0.0 – 39.6)	38.6 (23.6 – 77.1)	30.0 (30.0 – 38.6)	30.0 (0.0 – 36.4)

All values are medians (25^th^ – 75^th^ percentiles).

### Test-retest reliability and construct validity study

#### Test-retest reliability study

Table 3 shows the questionnaire items, their ICC values, and percentage agreement for all countries combined, for the test-retest reliability and construct validity study. Table 4 summarises the findings per category of the ENERGY-parent questionnaire. For the total sample across all countries, the test-retest reliability was good to excellent for 99.2% or all but one of the 121 response items. The test-retest reliability was comparable across the six countries.

**Table 3:** Agreement (per questionnaire item) between questionnaires (test-retest reliability) and questionnaire and interview responses (construct validity) as indicated by intraclass correlation coefficients (ICC) and percentage agreement (agree)

**Item**	** Reliability**		** Validity**	
	**ICC**	**agree**	**ICC**	**agree**
How many times a week on average do you drink soft drinks? (B1)	.87	77	.76	73
On a day that you drink soft drinks, how many glasses do you drink? (B2a)	.85	83	.61	71
On a day that you drink soft drinks, how many cans do you drink? (B2b)	.73	85	.38	74
On a day that you drink soft drinks, how many bottles do you drink? (B2c)	.68	91	.08	86
Drinking soft drinks is something I do without even really thinking about (B3)	.70	70	.37	47
There are soft drinks available at home for my child (B4)	.85	73	.67	65
I pay attention to the amount of soft drinks that my child drinks (B5)	.64	71	.61	65
If my child asks for soft drinks, I will give it to him/her (B6)	.79	75	.43	61
My child is allowed to take soft drinks whenever (s)he wants (B7)	.83	73	.64	59
I negotiate with my child how much soft drinks (s)he is allowed to drink (B8)	.69	62	.40	55
How often do you tell your child that soft drinks are not good for him/her? (B9)	.76	70	.60	66
How often do you tell your child that soft drinks can make him/her fat? (B10)	.84	70	.75	59
How often do you tell your child that soft drinks are bad for his/her teeth? (B11)	.78	70	.68	65
If I would like to drink soft drinks, I would restrain myself because of the presence of my child (B12)	.79	70	.50	53
If I prohibit my child from drinking soft drinks, (s)he tries to drink it anyway (B13)	.68	76	.78	81
If I prohibit my child from drinking soft drinks, I find it difficult to stick to my rule(s) if (s)he starts negotiating (B14)	.66	72	.65	77
I give soft drinks to my child as a reward or to comfort him/her (B15)	.75	86	.55	87
How often do you and/or your spouse/partner drink soft drinks together with your child? (B16)	.86	74	.67	61
If the price of soft drinks were double, my child would drink less soft drinks (B17)	.63	64	.46	58
On average, how much money do you give to your child to buy food and drinks per week? Please don’t include money you save or spend on clothes for your child (B18)	.80	88	.44	82
I would consider my child as being price conscious regarding food, snacks and beverages (B19)	.72	65	.71	59
I don’t give my child some foods, because they cost too much (B20)	.72	69	.68	61
What do you consider to be the three most important characteristics of your child’s meal during school hours? NUTRITIOUS (B21a)	.68	89	.61	89
What do you consider to be the three most important characteristics of your child’s meal during school hours? PROVIDES ENERGY (B21b)	.74	87	.56	78
What do you consider to be the three most important characteristics of your child’s meal during school hours? EXHIBITS HIGH VARIETY (B21c)	.75	88	.67	84
What do you consider to be the three most important characteristics of your child’s meal during school hours? SATISFIES MY CHILD’S LIKING (B21d)	.77	89	.59	80
What do you consider to be the three most important characteristics of your child’s meal during school hours? REASONABLE PRICE (B21e)	.79	94	.54	89
What do you consider to be the three most important characteristics of your child’s meal during school hours? HOME-PREPARED (B21f)	.83	92	.62	83
What do you consider to be the three most important characteristics of your child’s meal during school hours? ORGANIC (B21g)	.90	99	-.01	98
What do you consider to be the three most important characteristics of your child’s meal during school hours? VEGETARIAN (B21h)	.96	100	/	100
What do you consider to be the three most important characteristics of your child’s meal during school hours? TAKING INTO ACCOUNT RELIGIOUS REQUIREMENTS (B21i)	.92	100	1.00	100
How many times a week on average do you drink fruit juices? (C1)	.86	73	.89	70
On a day that you drink fruit juices, how many glasses do you drink? (C2a)	.77	78	.25	77
On a day that you drink fruit juices, how many cartons do you drink? (C2b)	.73	83	.38	86
Drinking fruit juices is something I do without really thinking about (C3)	.77	70	.56	56
There are fruit juices available at home for my child (C4)	.86	75	.63	61
I pay attention to the amount of fruit juices that my child drinks (C5)	.73	62	.54	57
If my child asks for fruit juices, I will give it to him/her (C6)	.79	72	.53	58
My child is allowed to take fruit juices whenever (s)he wants(C7)	.78	67	.59	48
I negotiate with my child how much fruit juices (s)he is allowed to drink (C8)	.68	62	.56	69
How often do you tell your child that fruit juices are not good for him/her?(C9)	.77	63	.76	62
How often do you tell your child that fruit juices can make him/her fat? (C10)	.83	72	.72	76
How often do you tell your child that fruit juices are bad for his/her teeth? (C11)	.76	70	.69	70
If I would like to drink fruit juices, I would restrain myself because of the presence of my child (C12)	.73	74	.38	74
If I prohibit my child from drinking fruit juices, (s)he tries to drink it anyway (C13)	.74	78	.38	78
If I prohibit my child from drinking fruit juices, I find it difficult to stick to my rule(s) if (s)he starts negotiating (C14)	.66	74	.68	75
I give fruit juices to my child as a reward or to comfort him/her (C15)	.78	83	.52	85
How often do you or your spouse/partner drink fruit juices together with your child? (C16)	.86	73	.68	67
From Monday to Friday, how many times do you usually eat breakfast? (D1)	.87	90	.89	90
How many times do you usually eat breakfast on the weekend? (D2)	.85	92	.82	94
Eating breakfast is something I do without even really thinking about (D3)	.78	71	.46	64
There are breakfast products (e.g. milk, cereal, bread) available at home for my child (D4)	.68	95	.28	98
I encourage my child to have breakfast (D5)	.62	80	.37	43
I pay attention what kind of products my child is eating for breakfast (D6)	.65	72	.37	68
My child is allowed to skip breakfast (D7)	.86	83	.57	66
I negotiate with my child on how much breakfast products (s)he is allowed to eat and/or drink (D8)	.74	66	.39	59
How often do you tell your child that eating breakfast is good for him/her? (D9)	.75	74	.75	57
If I prohibit my child from skipping breakfast, (s)he tries to skip it anyway (D10)	.60	74	.69	86
If I prohibit my child from skipping breakfast, I find it difficult to stick to my rule(s) if (s)he starts negotiating (D11)	.64	73	.74	74
I praise my child if (s)he eats breakfast (D12)	.86	67	.73	62
How often do you and/or your spouse/partner have breakfast together with your child? (D13)	.79	65	.80	71
How often do you and/or your spouse/partner have lunch together with your child? (D14)	.80	71	.77	77
How often do you and/or your spouse/partner have dinner together with your child? (D15)	.70	71	.78	79
I deliberately have smaller helpings as a means of controlling my weight (E1)	.81	70	.65	54
I do not eat certain foods because they make me fat (E2)	.73	67	.67	59
On a scale of 1 to 8, where 1 means no restraint in eating (eating as much as you want, whenever you want it) and 8 means total restraint (constantly limiting food intake and never “giving in”), what rating would you give yourself? (E3)	.80	72	.75	75
Do you have a paid job? (F1)	.94	98	.94	98
How many days do you usually travel by car to work? (F2)	.91	88	.91	80
How many days do you usually use public transport (bus, tram, metro) to go to work? (F3)	.92	97	.96	96
How many days do you usually cycle to work or to the public transport station? (F4)	.92	91	.91	87
If you cycle, how long does it take you to cycle to work or to the public transport station? (F5)	.76	83	.82	89
How many days a week do you usually walk to work or to the public transport station? (F6)	.95	94	.89	89
If you walk, how long does it take you to walk to work or to the public transport station? (F7)	.77	87	.46	81
About how many days a week do you usually participate in physical activities/sports in your leisure time? I DO NOT PARTICIPATE IN ANY PHYSICAL ACTIVITIES/SPORTS (F8a)	.75	90	.81	94
About how many days a week do you usually participate in physical activities/sports in your leisure time? WEEKDAYS (F8b)	.89	80	.83	77
About how many days a week do you usually participate in physical activities/sports in your leisure time? WEEKENDDAYS (F8c)	.79	87	.85	85
About how much time a week do you participate in physical activities/sports in your leisure time? WEEKDAYS (F9a)	.88	74	.88	75
About how much time a week do you participate in physical activities/sports in your leisure time? WEEKENDDAYS (F9b)	.85	84	.81	79
Physical activity is something that I do without really thinking about (F10)	.83	73	.47	44
I pay for my child to take part in physical activity/sports (F11)	.89	78	.77	77
I bring my child to physical activity/sport sessions (F12)	.83	75	.79	64
I encourage my child to take part in physical activity/sports (F13)	.80	78	.30	54
I pay attention that my child does enough physical activity/sports (F14)	.72	71	.51	62
My child is allowed to skip physical activity/sport sessions whenever (s)he wants (F15)	.69	78	.80	78
I negotiate with my child on how much physical activity/sports (s)he does (F16)	.69	64	.68	64
How often do you tell your child physical activity/sports are good for him/her? (F17)	.75	70	.56	66
If I try to prohibit my child from not taking part in physical activity/sport sessions, (s)he will try to skip it anyway (F18)	.55	70	.60	76
If I try to prohibit my child from skipping a physical activity/sport session, I find it difficult to stick to my rule(s) if (s)he starts negotiating (F19)	.72	74	.88	87
I praise my child if (s)he takes part in physical activity/sports (F20)	.72	68	.57	60
I punish my child by not allowing him/her taking part in his/her physical activity/sports (F21)	.67	81	.60	90
I set a time limit on how much time of physical activity/sports my child can do in order to devote more time to his/her homework or other important things (F22)	.81	69	.68	53
I do not allow my child to take part in physical activity/sports in his/her free time so (s)he can concentrate on his/her studies (F23)	.66	72	.74	69
How often do you/your spouse/partner participate in physical activity/sports together with your child? (F24)	.80	73	.56	57
I let my child participate in physical activity/sports lessons less than I like, because it is too expensive (F25)	.77	74	.66	71
About how many hours a day do you usually watch television (including DVDs and videos) in your free time? WEEKDAYS (G1a)	.79	60	.90	70
About how many hours a day do you usually watch television (including DVDs and videos) in your free time? WEEKENDDAYS (G1b)	.83	57	.73	61
About how many hours a day do you usually use your computer for activities like chatting online, internet, emailing or playing games on a computer, games console (e.g. Playstation, Xbox, GameCube) during leisure time? WEEKDAYS (G2a)	.83	73	.77	69
About how many hours a day do you usually use your computer for activities like chatting online, internet, emailing or playing games on a computer, games console (e.g. Playstation, Xbox, GameCube) during leisure time? WEEKENDDAYS (G2b)	.86	69	.77	75
About how many hours a day do you usually use your mobile phone for calling, texting, playing games or surfing on the internet during leisure time? WEEKDAYS (G3a)	.84	83	.72	80
About how many hours a day do you usually use your mobile phone for calling, texting, playing games or surfing on the internet during leisure time? WEEKENDDAYS (G3b)	.75	79	.51	82
Watching television is something I do without even really thinking about (G4)	.75	66	.46	44
In general, how often do you watch television during breakfast? (G5a)	.74	83	.69	78
In general, how often do you watch television during lunch? (G5b)	.87	81	.70	76
In general, how often do you watch television during dinner? (G5c)	.91	80	.79	75
TV/video/DVD is available in my child’s room (G6)	.97	99	.94	97
I pay attention to the amount of time my child watches TV/video/DVD (G7)	.70	72	.41	42
If my child asks if (s)he is allowed to watch television, I will allow it (G8)	.66	77	.61	58
My child is allowed to watch TV/video/DVD whenever (s)he wants (G9)	.72	61	.55	43
I negotiate with my child how much TV/video/DVD (s)he is allowed to watch (G10)	.73	64	.51	58
How often do you tell your child that watching TV/video/DVD is not good for him/her? (G11)	.76	69	.51	59
How often do you tell your child that watching TV/video/DVD can make him/her fat? (G12)	.83	73	.76	72
How often do you tell your child that watching TV/video/DVD is bad for his/her eyesight? (G13)	.82	70	.75	63
If I would like to watch TV/video/DVD, I would restrain myself because of the presence of my child (G14)	.76	71	.68	62
If I prohibit my child from watching TV/video/DVD, (s)he tries to do it anyway (G15)	.77	71	.71	77
If I prohibit my child from watching TV/video/DVD, I find it difficult to stick to my rule(s) if (s)he starts negotiating (G16)	.68	75	.71	77
I allow my child to watch TV/video/DVD as a reward or to comfort him/her (G17)	.78	74	.67	71
How often do you/your spouse/partner watch TV/video/DVD together with your child? (G18)	.72	69	.70	69
What do you think of your child’s weight? (H1)	.93	96	.83	94
Does your child have a set daily routine for bedtime? (H2)	.79	69	.42	95
How many hours of sleep does your child usually have during the night? WEEKDAYS (H3a)	.81	79	.80	76
How many hours of sleep does your child usually have during the night? WEEKENDDAYS (H3b)	.78	74	.79	70

Results are presented for all countries.

**Table 4:** Overview of the results per section of the ENERGY-parent questionnaire for both test-retest reliability and construct validity, combined for all countries

		**test-retest reliability**				**construct validity**				
**section of the ENERGY-parent questionnaire**	**# items**	**range**	**excellent n (%)**	**good n (%)**	**moderate n (%)**	**range**	**excellent n (%)**	**good n (%)**	**moderate n (%)**	**poor n (%)**
B1 - soft drinks	18	.64 - .87	7 (38.9)	11 (61.1)	-	.08 - .78	-	12 (66.7)	4 (22.2)	2 (11.1)
B2 - influence food choices	13	.63 - .96	5 (38.5)	8 (61.5)	-	.44 - 1.00	3 (23.1)	9 (69.2)	1 (7.7)	-
C - fruit juices	17	.66 - .86	4 (30.8)	13 (69.2)	-	.25 - .89	1 (5.9)	10 (58.9)	6 (35.3)	-
D - breakfast	15	.60 - .87	5 (33.3)	10 (66.6)	-	.28 - .89	3 (20.0)	7 (46.7)	3 (20.0)	2 (13.3)
E - dieting behaviour	3	.73 - .81	1 (33.3)	2 (66.6)	-	.65 - .75	-	3 (100)	-	-
F - physical activity behaviour	28	.55 - .95	13 (46.4)	14 (50.0)	1 (3.6)	.30 - .96	13 (46.4)	9 (32.1)	5 (17.9)	1 (3.6)
G - screen viewing behaviour	23	.66 - .97	9 (39.1)	14 (60.9)	-	.41 - .93	2 (8.7)	16 (69.6)	5 (21.7)	0 (0.0)
H - sleeping behaviour child	4	.78 - .81	2 (50.0)	2 (50.0)	-	.42 - .79	2 (50.0)	2 (50.0)	-	-
**Overall**	**121**	**.55 - .97**	**46 (38.0)**	**74 (61.2)**	**1 (0.8)**	**.08 - 1.00**	**24 (19.8)**	**68 (56.2)**	**24 (19.8)**	**5 (4.1)**

#### Construct validity study

Construct validity appeared to be good to excellent for 92 out of 121 items (76.0%). For the remaining 29 items, construct validity was moderate for 24 (19.8%) and poor for 5 (4.1%) items. Three response items did not show enough variability, resulting in ICCs ≤ .40, but had high (≥ 90%) percentage agreement (Table 4).

The construct validity was comparable across all countries, except for Belgium and Greece. The Belgian and Greek data showed lower construct validity values than the other countries, especially for the constructs that were already more likely to show moderate or poor values in the overall data set.

Country-specific values can be found in Additional file 2 and Additional file 3.

## Discussion

### General

The results of our study indicate that the ENERGY-parent questionnaire, assessing parenting practices, parental energy balance-related behaviours and their potential determinants, has adequate test-retest reliability and construct validity.

To our knowledge, this is the first study assessing the reliability and validity of a questionnaire on parental behaviours, their determinants and parenting practices for different EBRBs across different countries.

### Test-retest reliability

All but one item showed good to excellent test-retest reliability. A large majority of the items had high ICC values and percentage agreement. Some items (e.g. ‘*There are breakfast products (e.g. milk, cereal, bread) available at home for my child*’) had high agreement with low ICC values, due to little variation in parental responses. Despite these mixed ICC scores, the majority of the percentage agreement between the questionnaires indicated good to excellent test-retest reliability of the questions.

### Construct validity

Values for the construct validity were somewhat lower than those for the test-rest reliability. A closer examination indicated that all questions on automaticity (e.g. ‘*Drinking soft drinks is something I do without really thinking about*’) - often used in questionnaires to assess habit strength regarding a specific behaviour [10] - showed moderate or even poor values for validity. The lower validity for the habit strength questions is consistent with the findings of the ENERGY-child questionnaire [3]. These findings indicate that these questions should not be used in further research.

Also, with regard to parenting practices, some questions showed lower ICCs across EBRBs. The questions in which parents were asked to indicate whether they negotiate with their child about the EBRBs had moderate or poor construct validity. The interviews indicated that ‘negotiation with the child’ was a concept many respondents did not understand or find relevant. Consequently, these questions should not be used in future research.

The Greek data, and the Belgian data in particular, showed lower construct validity values than the other countries, especially for the constructs that were already more likely to show moderate or poor values in the overall data set. The reason for this deviation may be that in Belgium and Greece almost all interviews (i.e. 17 in Belgium and 15 in Greece) were conducted via telephone, whereas in the other countries the majority of the interviews were conducted face-to-face.

Since the number of respondents per country was relatively small (i.e. 20 in most countries), more value should be attached to the results of the overall data set than to the country-specific analyses.

## Conclusions

Our findings suggest that the ENERGY-parent questionnaire has good to excellent test-retest reliability and good construct validity among parents of European children aged 10–12 years.

Based on the results of the validity study, we strongly recommend adapting parts of the ENERGY-parent questionnaire if used in future research.

## Availability of supporting data

The data set supporting the results of this article are included within the article and its additional files.

## Abbreviations

ICC, Intra-class correlation coefficient; EBRB, Energy balance-related behaviours.

## Competing interests

There are no financial or non-financial competing interests to declare in relation to this manuscript.

## Authors’ contributions

ASS set up and coordinated the study, supervised data analyses and drafted the manuscript. MJMC supervised the study set up and helped to draft the manuscript. LU carried out the statistical analyses. FNV, WvL, JMFA, SS and YM collected the data in the countries or supervised data collection. MvS helped to develop the data collection protocol. CT advised on the statistical methods. JB is the coordinator of the ENERGY-project and helped to draft the manuscript. All co-authors read and approved the final version of the manuscript.

## Funding

The ENERGY-project is funded by the Seventh Framework Programme (CORDIS FP7) of the European Commission, HEALTH (*FP7-HEALTH-2007-B).* The content of this article reflects only the authors’ views and the European Community is not liable for any use that may be made of the information contained therein.

## Supplementary Material

Additional file 1 Additional information on the recruitment procedure and data management. Click here for file

Additional file 2 Table Country-specific results of the test-retest reliability study of the ENERGY-parent questionnaire: agreement (per questionnaire item) between questionnaires as indicated by intraclass correlation coefficients (ICC) and percentage agreement (agree). Click here for file

Additional file 3 Table Country-specific results of the construct validity study of the ENERGY-parent questionnaire: agreement (per questionnaire item) between questionnaire and interview as indicated by intraclass correlation coefficients (ICC) and percentage agreement (agree). Click here for file
