# Supplementary material for: Test-retest reliability and construct validity of the ENERGY-parent questionnaire on parenting practices, energy balance-related behaviours and their potential behavioural determinants: the ENERGY-project
Source: BMC Res Notes. 2012 Aug 13;5:434. doi: 10.1186/1756-0500-5-434 (PMC3490786; doi:10.1186/1756-0500-5-434)
Supplement: Additional file 1 — Additional information on the recruitment procedure and data management. [file 1756-0500-5-434-S1.doc]

**Additional file 1**

Additional information on the recruitment procedure and data management

***Recruitment procedure***

We informed parents about the aim and methods of the study by an information letter, which was handed to their children at school or directly to the parent. The information letter was accompanied by a reply card for participation. Parents who returned the card were contacted for further explanation of the study methods and to make an appointment to (1) fill in the questionnaire at baseline and exactly one week later (test-retest reliability study); or (2) to fill in the questionnaire and subsequently participate in a cognitive interview (construct validity study), preferably face-to-face, otherwise via telephone. Parents could indicate in which of the two studies they would like to participate, i.e. the test-retest reliability or the construct validity study.

In countries where ethical approval was necessary for such non-intervention studies this was obtained from the relevant ethical committee. (Belgium: Ethical Committee of Ghent University; Greece: Ethics Committee of Harokopio University of Athens; Hungary: Scientific and Research Ethical Committee of the Medical Scientific Council; the Netherlands: Medical Ethics Review Committee VU University Medical Center; Norway: Regional Ethical committee, south/east Norway; Spain: Comité Ético de Investigación Clínica de Aragón) and informed consent of the parents was obtained prior to the study (Hungary and Spain: active informed consent, other countries: no informed consent required).

***Data management***

A standard data management protocol was developed to ensure missing and ambiguous values were handled consistently.

- Double data entry

For both the test-retest reliability study and the validity study a randomly chosen 5% of the questionnaires were re-entered in SPSS (double data entry) to check for typing errors and misinterpretation. A difference of less than 3% was accepted. In case there was a difference of more than 3%, the cases had to be re-entered in the original data set and the procedure was repeated. Across the countries, the rate of disagreement in the test-retest reliability and construct validity ranged from 0.0% - 2.8% and 0.0% - 2.1%, respectively.

- Data definition

The data definition process consisted of adding variable labels, value labels and missing value definitions to the original data files.

- Data cleaning

During the data cleaning original data was checked for duplicate records, system-missing values, out-of-range values and logical inconsistencies.
