# Supplementary material for: Test-retest reliability and construct validity of the ENERGY-parent questionnaire on parenting practices, energy balance-related behaviours and their potential behavioural determinants: the ENERGY-project
Source: BMC Res Notes. 2012 Aug 13;5:434. doi: 10.1186/1756-0500-5-434 (PMC3490786; doi:10.1186/1756-0500-5-434)
Supplement: Additional file 2 — Table Country-specific results of the test-retest reliability study of the ENERGY-parent questionnaire: agreement (per questionnaire item) between questionnaires as indicated by intraclass correlation coefficients (ICC) and percentage agreement (agree). [file 1756-0500-5-434-S2.doc]

**Additional file 2.**

**Table.** Country-specific results of the test-retest reliability study of the ENERGY-parent questionnaire: agreement (per questionnaire item) between questionnaires as indicated by intraclass correlation coefficients (ICC) and percentage agreement (agree).

| **Country** | **Belgium** | | **Greece** | | **Hungary** | | **Netherlands** | | **Norway** | | **Spain** | |
| --- | --- | --- | --- | --- | --- | --- | --- | --- | --- | --- | --- | --- |
| Item | ICC | agree | ICC | agree | ICC | agree | ICC | agree | ICC | agree | ICC | agree |
| How many times a week on average do you drink soft drinks? (B1) | .72 | 65 | .92 | 72 | .98 | 80 | .93 | 83 | .72 | 77 | .87 | 72 |
| On a day that you drink soft drinks, how many glasses do you drink? (B2a) | .59 | 73 | .67 | 90 | .96 | 91 | .91 | 88 | .90 | 71 | .59 | 79 |
| On a day that you drink soft drinks, how many cans do you drink? (B2b) | .44 | 79 | .50 | 75 | .93 | 95 | .96 | 94 | .92 | 96 | .68 | 77 |
| On a day that you drink soft drinks, how many bottles do you drink? (B2c) | .35 | 88 | .37 | 91 | .88 | 89 | .97 | 98 | .75 | 93 | .43 | 87 |
| Drinking soft drinks is something I do without even really thinking about (B3) | .43 | 50 | .64 | 71 | .87 | 85 | .93 | 82 | .59 | 60 | .59 | 77 |
| There are soft drinks available at home for my child (B4) | .80 | 71 | .75 | 66 | .94 | 84 | .92 | 85 | .66 | 67 | .78 | 63 |
| I pay attention to the amount of soft drinks that my child drinks (B5) | .49 | 50 | .42 | 80 | .77 | 90 | .91 | 85 | .33 | 61 | .62 | 63 |
| If my child asks for soft drinks, I will give it to him/her (B6) | .75 | 75 | .74 | 73 | .95 | 92 | .93 | 85 | .70 | 67 | .55 | 55 |
| My child is allowed to take soft drinks whenever (s)he wants (B7) | .71 | 58 | .73 | 79 | .96 | 86 | .92 | 81 | .81 | 63 | .77 | 70 |
| I negotiate with my child how much soft drinks (s)he is allowed to drink (B8) | .50 | 36 | .60 | 71 | .87 | 82 | .87 | 83 | .66 | 49 | .57 | 54 |
| How often do you tell your child that soft drinks are not good for him/her? (B9) | .62 | 54 | .69 | 79 | .93 | 82 | .93 | 83 | .58 | 67 | .57 | 61 |
| How often do you tell your child that soft drinks can make him/her fat? (B10) | .71 | 52 | .78 | 75 | .91 | 88 | .93 | 83 | .78 | 69 | .74 | 57 |
| How often do you tell your child that soft drinks are bad for his/her teeth? (B11) | .49 | 51 | .84 | 77 | .96 | 90 | .94 | 81 | .65 | 63 | .67 | 55 |
| If I would like to drink soft drinks, I would restrain myself because of the presence of my child (B12) | .67 | 55 | .72 | 79 | .97 | 94 | .88 | 87 | .69 | 53 | .65 | 61 |
| If I prohibit my child from drinking soft drinks, (s)he tries to drink it anyway (B13) | .51 | 68 | .72 | 84 | .84 | 90 | .90 | 79 | .38 | 71 | .56 | 66 |
| If I prohibit my child from drinking soft drinks, I find it difficult to stick to my rule(s) if (s)he starts negotiating (B14) | .30 | 55 | .66 | 77 | .88 | 94 | .85 | 79 | .55 | 63 | .54 | 72 |
| I give soft drinks to my child as a reward or to comfort him/her (B15) | .53 | 81 | .90 | 96 | .87 | 96 | .97 | 92 | .63 | 82 | .42 | 69 |
| How often do you and/or your spouse/partner drink soft drinks together with your child? (B16) | .81 | 64 | .85 | 80 | .98 | 92 | .96 | 86 | .83 | 66 | .62 | 55 |
| If the price of soft drinks were double, my child would drink less soft drinks (B17) | .33 | 48 | .67 | 64 | .82 | 82 | .60 | 73 | .63 | 59 | .63 | 58 |
| On average, how much money do you give to your child to buy food and drinks per week? Please don’t include money you save or spend on clothes for your child (B18) | .70 | 90 | .95 | 89 | 1.00 | 100 | .92 | 90 | .60 | 8 | .54 | 78 |
| I would consider my child as being price conscious regarding food, snacks and beverages (B19) | .54 | 43 | .85 | 60 | .95 | 92 | .82 | 73 | .38 | 65 | .58 | 67 |
| I don’t give my child some foods, because they cost too much (B20) | .62 | 60 | .58 | 66 | .95 | 86 | .76 | 79 | .62 | 67 | .59 | 62 |
| What do you consider to be the three most important characteristics of your child’s meal during school hours? NUTRITIOUS (B21a) | .59 | 87 | .62 | 91 | .88 | 96 | .76 | 89 | .57 | 84 | .56 | 88 |
| What do you consider to be the three most important characteristics of your child’s meal during school hours? PROVIDES ENERGY (B21b) | .49 | 82 | .73 | 89 | .92 | 96 | .77 | 88 | .55 | 79 | .75 | 88 |
| What do you consider to be the three most important characteristics of your child’s meal during school hours? EXHIBITS HIGH VARIETY (B21c) | .58 | 84 | .57 | 89 | .92 | 96 | .73 | 87 | .60 | 80 | .78 | 90 |
| What do you consider to be the three most important characteristics of your child’s meal during school hours? SATISFIES MY CHILD’S LIKING (B21d) | .70 | 85 | .74 | 88 | .92 | 96 | .77 | 88 | .71 | 87 | .60 | 88 |
| What do you consider to be the three most important characteristics of your child’s meal during school hours? REASONABLE PRICE (B21e) | .51 | 82 | .88 | 98 | .91 | 96 | .90 | 96 | .00 | 97 | .79 | 98 |
| What do you consider to be the three most important characteristics of your child’s meal during school hours? HOME-PREPARED (B21f) | .66 | 99 | .91 | 96 | .81 | 92 | .90 | 96 | .78 | 92 | .72 | 90 |
| What do you consider to be the three most important characteristics of your child’s meal during school hours? ORGANIC (B21g) | .80 | 99 | 1.00 | 100 | 1.00 | 100 | .91 | 98 | / | 100 | .66 | 98 |
| What do you consider to be the three most important characteristics of your child’s meal during school hours? VEGETARIAN (B21h) | 1.00 | 100 | / | 100 | 1.00 | 100 | 1.00 | 100 | / | 100 | .85 | 98 |
| What do you consider to be the three most important characteristics of your child’s meal during school hours? TAKING INTO ACCOUNT RELIGIOUS REQUIREMENTS (B21i) | / | 100 | 1.00 | 100 | / | 100 | 1.00 | 100 | / | 100 | .88 | 98 |
| How many times a week on average do you drink fruit juices? (C1) | .75 | 59 | .96 | 77 | .93 | 94 | .91 | 86 | .82 | 74 | .74 | 47 |
| On a day that you drink fruit juices, how many glasses do you drink? (C2a) | .46 | 75 | .48 | 86 | .79 | 76 | .96 | 87 | .54 | 65 | .53 | 74 |
| On a day that you drink fruit juices, how many cartons do you drink? (C2b) | .21 | 89 | .47 | 69 | .86 | 82 | .77 | 88 | .79 | 92 | .25 | 76 |
| Drinking fruit juices is something I do without really thinking about (C3) | .77 | 69 | .72 | 58 | .86 | 89 | .96 | 86 | .74 | 44 | .53 | 68 |
| There are fruit juices available at home for my child (C4) | .74 | 60 | .83 | 79 | .95 | 90 | .91 | 85 | .83 | 71 | .86 | 71 |
| I pay attention to the amount of fruit juices that my child drinks (C5) | .58 | 46 | .76 | 61 | .91 | 88 | .92 | 75 | .77 | 57 | .42 | 48 |
| If my child asks for fruit juices, I will give it to him/her (C6) | .68 | 59 | .74 | 73 | .90 | 90 | .78 | 81 | .85 | 71 | .66 | 61 |
| My child is allowed to take fruit juices whenever (s)he wants(C7) | .64 | 49 | .66 | 73 | .96 | 90 | .83 | 75 | .82 | 56 | .59 | 58 |
| I negotiate with my child how much fruit juices (s)he is allowed to drink (C8) | .43 | 52 | .74 | 55 | .83 | 82 | .85 | 75 | .61 | 53 | .58 | 56 |
| How often do you tell your child that fruit juices are not good for him/her?(C9) | .60 | 54 | .63 | 59 | .89 | 78 | .76 | 71 | .71 | 64 | .60 | 55 |
| How often do you tell your child that fruit juices can make him/her fat? (C10) | .77 | 59 | .89 | 73 | .96 | 96 | .83 | 77 | .68 | 76 | .65 | 58 |
| How often do you tell your child that fruit juices are bad for his/her teeth? (C11) | .70 | 65 | .68 | 57 | .92 | 88 | .86 | 77 | .66 | 62 | .70 | 69 |
| If I would like to drink fruit juices, I would restrain myself because of the presence of my child (C12) | .61 | 56 | .72 | 66 | .94 | 94 | .83 | 83 | .72 | 66 | .48 | 87 |
| If I prohibit my child from drinking fruit juices, (s)he tries to drink it anyway (C13) | .64 | 73 | .68 | 74 | .79 | 90 | .85 | 79 | .30 | 70 | .92 | 85 |
| If I prohibit my child from drinking fruit juices, I find it difficult to stick to my rule(s) if (s)he starts negotiating (C14) | .39 | 61 | .58 | 69 | .92 | 90 | .88 | 82 | .18 | 74 | .79 | 74 |
| I give fruit juices to my child as a reward or to comfort him/her (C15) | .43 | 78 | .83 | 75 | .95 | 94 | .93 | 88 | .66 | 84 | .78 | 81 |
| How often do you or your spouse/partner drink fruit juices together with your child? (C16) | .81 | 66 | .84 | 62 | .96 | 96 | .95 | 85 | .84 | 68 | .78 | 65 |
| From Monday to Friday, how many times do you usually eat breakfast? (D1) | .87 | 94 | .77 | 79 | .96 | 92 | .99 | 98 | .73 | 87 | .87 | 92 |
| How many times do you usually eat breakfast on the weekend? (D2) | .79 | 93 | .86 | 80 | .79 | 96 | .97 | 98 | .49 | 95 | .39 | 90 |
| Eating breakfast is something I do without even really thinking about (D3) | .66 | 65 | .66 | 55 | .81 | 90 | .90 | 71 | .66 | 71 | .85 | 81 |
| There are breakfast products (e.g. milk, cereal, bread) available at home for my child (D4) | -.04 | 90 | .73 | 89 | 1.00 | 100 | .67 | 96 | / | 100 | .81 | 96 |
| I encourage my child to have breakfast (D5) | .50 | 73 | .35 | 71 | .92 | 93 | .83 | 89 | .56 | 81 | .25 | 75 |
| I pay attention what kind of products my child is eating for breakfast (D6) | .58 | 49 | .70 | 82 | .82 | 92 | .71 | 79 | .60 | 62 | .39 | 70 |
| My child is allowed to skip breakfast (D7) | .83 | 80 | .85 | 77 | .96 | 94 | .92 | 88 | .81 | 87 | .48 | 74 |
| I negotiate with my child on how much breakfast products (s)he is allowed to eat and/or drink (D8) | .61 | 50 | .70 | 68 | .91 | 90 | .77 | 73 | .52 | 57 | .82 | 64 |
| How often do you tell your child that eating breakfast is good for him/her? (D9) | .57 | 52 | .74 | 82 | .78 | 90 | .89 | 77 | .64 | 78 | .40 | 75 |
| If I prohibit my child from skipping breakfast, (s)he tries to skip it anyway (D10) | .27 | 64 | .41 | 57 | .86 | 90 | .79 | 83 | .64 | 73 | .66 | 81 |
| If I prohibit my child from skipping breakfast, I find it difficult to stick to my rule(s) if (s)he starts negotiating (D11) | .26 | 66 | .53 | 54 | .88 | 90 | .83 | 81 | .59 | 69 | .78 | 79 |
| I praise my child if (s)he eats breakfast (D12) | .62 | 50 | .68 | 67 | .86 | 86 | .92 | 75 | .73 | 56 | .91 | 76 |
| How often do you and/or your spouse/partner have breakfast together with your child? (D13) | .64 | 55 | .92 | 84 | .92 | 92 | .86 | 79 | .50 | 78 | .71 | 53 |
| How often do you and/or your spouse/partner have lunch together with your child? (D14) | .62 | 66 | .94 | 82 | .98 | 98 | .92 | 81 | .17 | 51 | .76 | 70 |
| How often do you and/or your spouse/partner have dinner together with your child? (D15) | .55 | 72 | .89 | 80 | .90 | 94 | .78 | 89 | .33 | 72 | .63 | 60 |
| I deliberately have smaller helpings as a means of controlling my weight (E1) | .77 | 63 | .83 | 64 | .88 | 82 | .85 | 75 | .60 | 68 | .83 | 70 |
| I do not eat certain foods because they make me fat (E2) | .55 | 57 | .82 | 64 | .83 | 86 | .88 | 77 | .61 | 62 | .63 | 57 |
| On a scale of 1 to 8, where 1 means no restraint in eating (eating as much as you want, whenever you want it) and 8 means total restraint (constantly limiting food intake and never “giving in”), what rating would you give yourself? (E3) | .76 | 68 | .82 | 70 | .86 | 80 | .97 | 81 | .50 | 70 | .74 | 61 |
| Do you have a paid job? (F1) | .92 | 97 | 1.00 | 100 | .98 | 98 | .93 | 98 | .70 | 97 | .97 | 98 |
| How many days do you usually travel by car to work? (F2) | .89 | 86 | .80 | 92 | .90 | 91 | .99 | 92 | .81 | 81 | .89 | 88 |
| How many days do you usually use public transport (bus, tram, metro) to go to work? (F3) | 1.00 | 100 | .99 | 96 | .87 | 94 | 1.00 | 100 | .37 | 94 | .84 | 91 |
| How many days do you usually cycle to work or to the public transport station? (F4) | .98 | 91 | .49 | 96 | .95 | 89 | 1.00 | 98 | .69 | 78 | .80 | 88 |
| If you cycle, how long does it take you to cycle to work or to the public transport station? (F5) | .83 | 91 | .39 | 87 | 1.00 | 100 | .92 | 92 | .55 | 77 | .54 | 39 |
| How many days a week do you usually walk to work or to the public transport station? (F6) | .88 | 93 | 1.00 | 98 | .93 | 94 | 1.00 | 98 | .70 | 89 | .98 | 91 |
| If you walk, how long does it take you to walk to work or to the public transport station? (F7) | .66 | 91 | .85 | 91 | .99 | 97 | .67 | 88 | .60 | 78 | .86 | 66 |
| About how many days a week do you usually participate in physical activities/sports in your leisure time? I DO NOT PARTICIPATE IN ANY PHYSICAL ACTIVITIES/SPORTS (F8a) | .82 | 91 | .70 | 85 | .95 | 98 | .79 | 92 | .29 | 87 | .47 | 81 |
| About how many days a week do you usually participate in physical activities/sports in your leisure time? WEEKDAYS (F8b) | .93 | 76 | .95 | 84 | .91 | 97 | .89 | 80 | .68 | 70 | .86 | 76 |
| About how many days a week do you usually participate in physical activities/sports in your leisure time? WEEKENDDAYS (F8c) | .79 | 88 | .61 | 81 | .59 | 85 | .91 | 90 | .84 | 94 | .77 | 80 |
| About how much time a week do you participate in physical activities/sports in your leisure time? WEEKDAYS (F9a) | .78 | 69 | .98 | 81 | .94 | 92 | .95 | 81 | .60 | 56 | .82 | 58 |
| About how much time a week do you participate in physical activities/sports in your leisure time? WEEKENDDAYS (F9b) | .70 | 65 | .94 | 75 | .95 | 89 | .93 | 92 | .73 | 50 | .46 | 70 |
| Physical activity is something that I do without really thinking about (F10) | .83 | 69 | .69 | 63 | .95 | 87 | .95 | 87 | .67 | 59 | .77 | 71 |
| I pay for my child to take part in physical activity/sports (F11) | .88 | 77 | .71 | 65 | .99 | 96 | .85 | 87 | .87 | 76 | .86 | 65 |
| I bring my child to physical activity/sport sessions (F12) | .74 | 67 | .81 | 81 | .85 | 90 | .83 | 83 | .82 | 64 | .82 | 67 |
| I encourage my child to take part in physical activity/sports (F13) | .79 | 67 | .76 | 77 | .82 | 86 | .89 | 83 | .67 | 79 | .72 | 82 |
| I pay attention that my child does enough physical activity/sports (F14) | .44 | 53 | .84 | 82 | .73 | 84 | .82 | 81 | .67 | 63 | .67 | 72 |
| My child is allowed to skip physical activity/sport sessions whenever (s)he wants (F15) | .11 | 84 | .54 | 72 | .91 | 90 | .90 | 90 | .75 | 64 | .49 | 58 |
| I negotiate with my child on how much physical activity/sports (s)he does (F16) | .46 | 53 | .86 | 65 | .94 | 90 | .69 | 77 | .61 | 50 | .54 | 49 |
| How often do you tell your child physical activity/sports are good for him/her? (F17) | .57 | 60 | .64 | 71 | .87 | 90 | .90 | 73 | .47 | 59 | .61 | 67 |
| If I try to prohibit my child from not taking part in physical activity/sport sessions, (s)he will try to skip it anyway (F18) | .20 | 59 | .68 | 65 | .88 | 88 | .73 | 79 | .68 | 65 | .21 | 33 |
| If I try to prohibit my child from skipping a physical activity/sport session, I find it difficult to stick to my rule(s) if (s)he starts negotiating (F19) | .37 | 67 | .70 | 63 | .95 | 90 | .73 | 83 | .63 | 66 | .62 | 76 |
| I praise my child if (s(he) takes part in physical activity/sports (F20) | .59 | 53 | .59 | 75 | .93 | 88 | .87 | 77 | .58 | 57 | .34 | 61 |
| I punish my child by not allowing him/her taking part in his/her physical activity/sports (F21) | .44 | 66 | .65 | 78 | .91 | 98 | .91 | 79 | .65 | 95 | .46 | 72 |
| I set a time limit on how much time of physical activity/sports my child can do in order to devote more time to his/her homework or other important things (F22) | .50 | 46 | .78 | 71 | .88 | 88 | .92 | 82 | .67 | 64 | .76 | 68 |
| I do not allow my child to take part in physical activity/sports in his/her free time so (s)he can concentrate on his/her studies (F23) | .43 | 53 | .45 | 60 | .59 | 92 | .92 | 88 | .36 | 90 | .76 | 61 |
| How often do you/your spouse/partner participate in physical activity/sports together with your child? (F24) | .53 | 61 | .75 | 69 | .93 | 86 | .94 | 92 | .60 | 59 | .82 | 64 |
| I let my child participate in physical activity/sports lessons less than I like, because it is too expensive (F25) | .53 | 59 | .76 | 71 | .95 | 88 | .93 | 85 | .67 | 82 | .60 | 69 |
| About how many hours a day do you usually watch television (including DVDs and videos) in your free time? WEEKDAYS (G1a) | .80 | 64 | .92 | 70 | .95 | 82 | .98 | 85 | .45 | 10 | .33 | 38 |
| About how many hours a day do you usually watch television (including DVDs and videos) in your free time? WEEKENDDAYS (G1b) | .79 | 52 | .86 | 52 | .97 | 98 | .88 | 62 | .73 | 46 | .66 | 41 |
| About how many hours a day do you usually use your computer for activities like chatting online, internet, emailing or playing games on a computer, games console (e.g.Playstation, Xbox, GameCube) during leisure time? WEEKDAYS (G2a) | .67 | 70 | .81 | 73 | .89 | 84 | .97 | 81 | .84 | 67 | .75 | 62 |
| About how many hours a day do you usually use your computer for activities like chatting online, internet, emailing or playing games on a computer, games console (e.g.Playstation, Xbox, GameCube) during leisure time? WEEKENDDAYS (G2b) | .76 | 66 | .93 | 73 | .89 | 80 | .95 | 61 | .77 | 53 | .74 | 53 |
| About how many hours a day do you usually use your mobile phone for calling, texting, playing games or surfing on the internet during leisure time? WEEKDAYS (G3a) | .77 | 72 | .81 | 84 | .98 | 92 | .82 | 88 | .77 | 84 | .68 | 78 |
| About how many hours a day do you usually use your mobile phone for calling, texting, playing games or surfing on the internet during leisure time? WEEKENDDAYS (G3b) | .81 | 77 | .61 | 78 | .96 | 94 | .89 | 87 | .26 | 65 | .36 | 69 |
| Watching television is something I do without even really thinking about (G4) | .63 | 61 | .63 | 50 | .93 | 94 | .84 | 71 | .63 | 62 | .59 | 58 |
| In general, how often do you watch television during breakfast? (G5a) | .27 | 93 | .78 | 64 | .91 | 92 | .86 | 85 | .69 | 79 | .54 | 82 |
| In general, how often do you watch television during lunch? (G5b) | .57 | 82 | .78 | 64 | .96 | 94 | .93 | 90 | .74 | 86 | .83 | 64 |
| In general, how often do you watch television during dinner? (G5c) | .88 | 79 | .88 | 72 | .91 | 88 | .90 | 89 | .84 | 81 | .87 | 69 |
| TV/video/DVD is available in my child’s room (G6) | .94 | 99 | 1.00 | 100 | 1.00 | 100 | 1.00 | 100 | 1.00 | 100 | .76 | 93 |
| I pay attention to the amount of time my child watches TV/video/DVD (G7) | .51 | 50 | .57 | 71 | .85 | 90 | .77 | 83 | .73 | 69 | .80 | 79 |
| If my child asks if (s)he is allowed to watch television, I will allow it (G8) | .70 | 79 | .43 | 61 | .92 | 94 | .64 | 85 | .55 | 67 | .56 | 74 |
| My child is allowed to watch TV/video/DVD whenever (s)he wants (G9) | .72 | 54 | .66 | 49 | .86 | 80 | .74 | 73 | .58 | 53 | .65 | 57 |
| I negotiate with my child how much TV/video/DVD (s)he is allowed to watch (G10) | .66 | 44 | .63 | 70 | .91 | 86 | .84 | 67 | .76 | 76 | .55 | 52 |
| How often do you tell your child that watching TV/video/DVD is not good for him/her? (G11) | .54 | 54 | .44 | 62 | .95 | 92 | .90 | 86 | .62 | 59 | .72 | 66 |
| How often do you tell your child that watching TV/video/DVD can make him/her fat? (G12) | .60 | 59 | .89 | 77 | .97 | 90 | .80 | 67 | .90 | 92 | .75 | 65 |
| How often do you tell your child that watching TV/video/DVD is bad for his/her eyesight? (G13) | .57 | 52 | .78 | 68 | .88 | 88 | .93 | 87 | .76 | 72 | .63 | 57 |
| If I would like to watch TV/video/DVD, I would restrain myself because of the presence of my child (G14) | .58 | 62 | .79 | 66 | .85 | 88 | .85 | 85 | .61 | 59 | .66 | 66 |
| If I prohibit my child from watching TV/video/DVD, (s)he tries to do it anyway (G15) | .74 | 71 | .66 | 59 | .87 | 86 | .78 | 79 | .56 | 67 | .83 | 66 |
| If I prohibit my child from watching TV/video/DVD, I find it difficult to stick to my rule(s) if (s)he starts negotiating (G16) | .47 | 64 | .54 | 74 | .94 | 94 | .79 | 82 | .59 | 62 | .62 | 58 |
| I allow my child to watch TV/video/DVD as a reward or to comfort him/her (G17) | .77 | 74 | .73 | 70 | .98 | 96 | .86 | 73 | .71 | 62 | .61 | 64 |
| How often do you/r partner watch TV/video/DVD together with your child? (G18) | .72 | 63 | .73 | 68 | .97 | 90 | .92 | 85 | .27 | 46 | .51 | 53 |
| What do you think of your child’s weight? (H1) | .94 | 96 | .88 | 94 | 1.00 | 100 | .92 | 92 | .90 | 97 | .95 | 94 |
| Does your child have a set daily routine for bedtime? (H2) | .78 | 96 | .67 | 93 | 1.00 | 100 | .85 | 98 | 1.00 | 100 | .64 | 94 |
| How many hours of sleep does your child usually have during the night? WEEKDAYS (H3a) | .59 | 64 | .58 | 76 | .94 | 94 | .89 | 85 | .72 | 78 | .90 | 84 |
| How many hours of sleep does your child usually have during the night? WEEKENDDAYS (H3b) | .68 | 66 | .68 | 70 | .96 | 94 | .89 | 79 | .65 | 72 | .76 | 61 |
