# Supplementary material for: Test-retest reliability and construct validity of the ENERGY-parent questionnaire on parenting practices, energy balance-related behaviours and their potential behavioural determinants: the ENERGY-project
Source: BMC Res Notes. 2012 Aug 13;5:434. doi: 10.1186/1756-0500-5-434 (PMC3490786; doi:10.1186/1756-0500-5-434)
Supplement: Additional file 3 — Table Country-specific results of the construct validity study of the ENERGY-parent questionnaire: agreement (per questionnaire item) between questionnaire and interview as indicated by intraclass correlation coefficients (ICC) and percentage agreement (agree). [file 1756-0500-5-434-S3.doc]

**Additional file 3.**

**Table.** Country-specific results of the construct validity study of the ENERGY-parent questionnaire: agreement (per questionnaire item) between questionnaire and interview as indicated by intraclass correlation coefficients (ICC) and percentage agreement (agree).

| **Item** | **Belgium** | | **Greece** | | **Hungary** | | **Netherlands** | | **Norway** | | **Spain** | |
| --- | --- | --- | --- | --- | --- | --- | --- | --- | --- | --- | --- | --- |
|  | ICC | agree | ICC | agree | ICC | agree | ICC | agree | ICC | agree | ICC | agree |
| How many times a week on average do you drink soft drinks? (B1) | .71 | 37 | .98 | 90 | .66 | 65 | .74 | 79 | .83 | 85 | .97 | 78 |
| On a day that you drink soft drinks, how many glasses do you drink? (B2a) | .60 | 63 | .75 | 81 | .60 | 25 | .23 | 79 | .87 | 83 | .35 | 67 |
| On a day that you drink soft drinks, how many cans do you drink? (B2b) | .07 | 44 | .76 | 81 | / | 100 | .00 | 90 | .00 | 75 | 1.00 | 100 |
| On a day that you drink soft drinks, how many bottles do you drink? (B2c) | -.06 | 67 | / | 100 | -.50 | 25 | .00 | 95 | .81 | 92 | .00 | 100 |
| Drinking soft drinks is something I do without even really thinking about (B3) | -.08 | .30 | .78 | 64 | .39 | 30 | .31 | 54 | .15 | 33 | .00 | 83 |
| There are soft drinks available at home for my child (B4) | .26 | 30 | .93 | 85 | .61 | 65 | .52 | 69 | .68 | 74 | .78 | 67 |
| I pay attention to the amount of soft drinks that my child drinks (B5) | .21 | 25 | .52 | 90 | .64 | 60 | .78 | 61 | .86 | 85 | .91 | 75 |
| If my child asks for soft drinks, I will give it to him/her (B6) | .50 | 50 | .57 | 74 | .50 | 65 | .23 | 65 | .12 | 65 | .48 | 56 |
| My child is allowed to take soft drinks whenever (s)he wants (B7) | .63 | 45 | .81 | 80 | .74 | 55 | .36 | 58 | .63 | 35 | .72 | 67 |
| I negotiate with my child how much soft drinks (s)he is allowed to drink (B8) | .14 | 40 | .97 | 84 | .49 | 35 | .20 | 42 | .48 | 79 | -.32 | 44 |
| How often do you tell your child that soft drinks are not good for him/her? (B9) | .41 | 55 | .96 | 90 | .44 | 61 | .19 | 39 | .62 | 80 | .69 | 67 |
| How often do you tell your child that soft drinks can make him/her fat? (B10) | .43 | 30 | .90 | 75 | .60 | 42 | .62 | 61 | .94 | 94 | .74 | 44 |
| How often do you tell your child that soft drinks are bad for his/her teeth? (B11) | .43 | 40 | .89 | 85 | .33 | 63 | .82 | 53 | .70 | 80 | .67 | 67 |
| If I would like to drink soft drinks, I would restrain myself because of the presence of my child (B12) | .16 | 15 | .84 | 69 | .29 | 45 | .00 | 50 | .97 | 90 | .58 | 44 |
| If I prohibit my child from drinking soft drinks, (s)he tries to drink it anyway (B13) | .14 | 65 | .97 | 90 | .86 | 95 | .46 | 72 | 1.00 | 100 | .63 | 78 |
| If I prohibit my child from drinking soft drinks, I find it difficult to stick to my rule(s) if (s)he starts negotiating (B14) | .14 | 50 | .96 | 95 | .82 | 69 | .82 | 80 | .88 | 93 | .69 | 89 |
| I give soft drinks to my child as a reward or to comfort him/her (B15) | .22 | 80 | .84 | 95 | .66 | 90 | .58 | 79 | .90 | 90 | .00 | 44 |
| How often do you and/or your spouse/partner drink soft drinks together with your child? (B16) | .57 | 10 | .98 | 94 | .71 | 65 | .54 | 59 | .78 | 85 | .57 | 44 |
| If the price of soft drinks were double, my child would drink less soft drinks (B17) | .03 | 15 | .58 | 75 | .89 | 78 | .51 | 53 | .77 | 75 | .12 | 44 |
| On average, how much money do you give to your child to buy food and drinks per week? Please don’t include money you save or spend on clothes for your child (B18) | .21 | 74 | .88 | 90 | .72 | 74 | .02 | 89 | .90 | 89 | .52 | 63 |
| I would consider my child as being price conscious regarding food, snacks and beverages (B19) | .27 | 20 | .94 | 80 | .86 | 72 | .62 | 35 | .93 | 84 | .76 | 67 |
| I don’t give my child some foods, because they cost too much (B20) | .31 | 21 | .85 | 82 | .87 | 79 | .62 | 56 | .77 | 70 | .53 | 56 |
| What do you consider to be the three most important characteristics of your child’s meal during school hours? NUTRITIOUS (B21a) | .66 | 95 | .78 | 95 | -.12 | 73 | .42 | 80 | 1.00 | 100 | .74 | 89 |
| What do you consider to be the three most important characteristics of your child’s meal during school hours? PROVIDES ENERGY (B21b) | .50 | 85 | .75 | 90 | -.17 | 40 | .41 | 70 | .81 | 90 | .79 | 89 |
| What do you consider to be the three most important characteristics of your child’s meal during school hours? EXHIBITS HIGH VARIETY (B21c) | .46 | 75 | / | 100 | .22 | 60 | .72 | 85 | 1.00 | 100 | -.19 | 67 |
| What do you consider to be the three most important characteristics of your child’s meal during school hours? SATISFIES MY CHILD’S LIKING (B21d) | -.09 | 50 | 1.00 | 100 | .30 | 67 | .71 | 85 | .88 | 95 | .00 | 100 |
| What do you consider to be the three most important characteristics of your child’s meal during school hours? REASONABLE PRICE (B21e) | .64 | 85 | / | 100 | .13 | 60 | .84 | 95 | .00 | 95 | .00 | 89 |
| What do you consider to be the three most important characteristics of your child’s meal during school hours? HOME-PREPARED (B21f) | .00 | 90 | .86 | 95 | .21 | 67 | .46 | 75 | .63 | 90 | .32 | 67 |
| What do you consider to be the three most important characteristics of your child’s meal during school hours? ORGANIC (B21g) | / | 100 | / | 100 | .00 | 93 | .00 | 95 | / | 100 | / | 100 |
| What do you consider to be the three most important characteristics of your child’s meal during school hours? VEGETARIAN (B21h) | / | 100 | / | 100 | / | 100 | / | 100 | / | 100 | / | 100 |
| What do you consider to be the three most important characteristics of your child’s meal during school hours? TAKING INTO ACCOUNT RELIGIOUS REQUIREMENTS (B21i) | / | 100 | 1.00 | 100 | / | 100 | / | 100 | / | 100 | / | 100 |
| How many times a week on average do you drink fruit juices? (C1) | .87 | 50 | .94 | 85 | .93 | 60 | .72 | 74 | .86 | 75 | .94 | 78 |
| On a day that you drink fruit juices, how many glasses do you drink? (C2a) | .08 | 50 | .39 | 84 | .00 | 60 | .20 | 66 | .73 | 89 | .00 | 88 |
| On a day that you drink fruit juices, how many cartons do you drink? (C2b) | -.14 | 78 | / | 100 | .09 | 0 | .00 | 95 | .00 | 89 | .00 | 83 |
| Drinking fruit juices is something I do without really thinking about (C3) | .19 | 25 | .91 | 83 | .94 | 87 | -.12 | 20 | .70 | 55 | .45 | 57 |
| There are fruit juices available at home for my child (C4) | .20 | 40 | .77 | 90 | .64 | 40 | .66 | 42 | .79 | 85 | .67 | 78 |
| I pay attention to the amount of fruit juices that my child drinks (C5) | -.03 | 15 | .71 | 85 | .61 | 47 | .42 | 45 | .71 | 85 | .56 | 67 |
| If my child asks for fruit juices, I will give it to him/her (C6) | .27 | 40 | .37 | 75 | .78 | 55 | .44 | 53 | .71 | 75 | .45 | 44 |
| My child is allowed to take fruit juices whenever (s)he wants(C7) | .59 | 40 | .90 | 75 | / | / | .35 | 40 | .13 | 35 | .53 | 56 |
| I negotiate with my child how much fruit juices (s)he is allowed to drink (C8) | .25 | 37 | .84 | 85 | .50 | 85 | .06 | 50 | .96 | 95 | .79 | 44 |
| How often do you tell your child that fruit juices are not good for him/her?(C9) | .25 | 33 | .95 | 83 | / | / | .73 | 60 | / | / | .79 | 86 |
| How often do you tell your child that fruit juices can make him/her fat? (C10) | .27 | 65 | .53 | 84 | / | / | .84 | 70 | / | / | 1.00 | 100 |
| How often do you tell your child that fruit juices are bad for his/her teeth? (C11) | .32 | 55 | .93 | 90 | / | / | .65 | 60 | / | / | .88 | 86 |
| If I would like to drink fruit juices, I would restrain myself because of the presence of my child (C12) | .22 | 65 | .23 | 85 | .00 | 70 | .18 | 60 | .94 | 90 | .66 | 78 |
| If I prohibit my child from drinking fruit juices, (s)he tries to drink it anyway (C13) | .16 | 67 | .91 | 92 | .21 | 80 | .30 | 78 | .88 | 90 | .09 | 56 |
| If I prohibit my child from drinking fruit juices, I find it difficult to stick to my rule(s) if (s)he starts negotiating (C14) | .22 | 53 | .81 | 58 | .28 | 84 | .62 | 83 | .86 | 90 | .25 | 88 |
| I give fruit juices to my child as a reward or to comfort him/her (C15) | -.07 | 75 | .72 | 79 | .51 | 90 | .00 | 95 | .84 | 85 | .00 | 89 |
| How often do you or your spouse/partner drink fruit juices together with your child? (C16) | .11 | 25 | .91 | 90 | .84 | 60 | .62 | 74 | .91 | 90 | .86 | 63 |
| From Monday to Friday, how many times do you usually eat breakfast? (D1) | .88 | 100 | .98 | 95 | .81 | 75 | .00 | 95 | .00 | 95 | / | 100 |
| How many times do you usually eat breakfast on the weekend? (D2) | .00 | 95 | 1.00 | 100 | .80 | 80 | .00 | 95 | .00 | 95 | / | 100 |
| Eating breakfast is something I do without even really thinking about (D3) | .45 | 60 | .70 | 60 | .84 | 82 | .17 | 42 | .48 | 70 | .02 | 78 |
| There are breakfast products (e.g. milk, cereal, bread) available at home for my child (D4) | .00 | 95 | / | 100 | 1.00 | 100 | / | 100 | .00 | 95 | / | 100 |
| I encourage my child to have breakfast (D5) | .09 | 15 | .86 | 75 | .39 | 70 | .22 | 32 | .20 | 25 | -.15 | 33 |
| I pay attention what kind of products my child is eating for breakfast (D6) | .37 | 53 | .00 | 90 | .48 | 75 | .44 | 58 | .33 | 65 | -.15 | 63 |
| My child is allowed to skip breakfast (D7) | .08 | 70 | .63 | 70 | .69 | 68 | .36 | 72 | .38 | 60 | -.23 | 44 |
| I negotiate with my child on how much breakfast products (s)he is allowed to eat and/or drink (D8) | .37 | 37 | .50 | 63 | .68 | 70 | .17 | 60 | .37 | 70 | .19 | 44 |
| How often do you tell your child that eating breakfast is good for him/her? (D9) | .64 | 37 | .81 | 80 | .64 | 53 | .75 | 50 | .65 | 60 | .49 | 67 |
| If I prohibit my child from skipping breakfast, (s)he tries to skip it anyway (D10) | .01 | 63 | .93 | 89 | 1.00 | 100 | .50 | 91 | 1.00 | 100 | .00 | 63 |
| If I prohibit my child from skipping breakfast, I find it difficult to stick to my rule(s) if (s)he starts negotiating (D11) | .52 | 68 | .85 | 69 | .64 | 85 | / | 100 | .76 | 63 | .00 | 75 |
| I praise my child if (s)he eats breakfast (D12) | .46 | 30 | .82 | 84 | .62 | 50 | .70 | 58 | .90 | 90 | .80 | 56 |
| How often do you and/or your spouse/partner have breakfast together with your child? (D13) | .40 | 45 | 1.00 | 100 | .95 | 85 | .34 | 50 | .79 | 75 | .86 | 67 |
| How often do you and/or your spouse/partner have lunch together with your child? (D14) | .00 | 65 | .97 | 90 | .81 | 75 | .26 | 69 | .84 | 90 | .75 | 67 |
| How often do you and/or your spouse/partner have dinner together with your child? (D15) | .36 | 65 | .92 | 90 | .75 | 65 | .82 | 85 | .86 | 85 | .88 | 88 |
| I deliberately have smaller helpings as a means of controlling my weight (E1) | .64 | 40 | .72 | 45 | .72 | 60 | .36 | 30 | 1.00 | 100 | .55 | 44 |
| I do not eat certain foods because they make me fat (E2) | .30 | 42 | .85 | 60 | .78 | 70 | .37 | 25 | .91 | 85 | .92 | 78 |
| On a scale of 1 to 8, where 1 means no restraint in eating (eating as much as you want, whenever you want it) and 8 means total restraint (constantly limiting food intake and never “giving in”), what rating would you give yourself? (E3) | .80 | 68 | .93 | 70 | .60 | 78 | .05 | 29 | 1.00 | 100 | .48 | 78 |
| Do you have a paid job? (F1) | 1.00 | 100 | 1.00 | 100 | 1.00 | 100 | 1.00 | 100 | / | 100 | .76 | 78 |
| How many days do you usually travel by car to work? (F2) | .67 | 63 | .99 | 94 | .79 | 77 | .92 | 72 | .92 | 85 | 1.00 | 100 |
| How many days do you usually use public transport (bus, tram, metro) to go to work? (F3) | / | 100 | 1.00 | 100 | .98 | 85 | .92 | 95 | .46 | 95 | / | 100 |
| How many days do you usually cycle to work or to the public transport station? (F4) | .03 | 90 | .92 | 94 | .99 | 92 | .97 | 84 | .94 | 80 | .00 | 86 |
| If you cycle, how long does it take you to cycle to work or to the public transport station? (F5) | .00 | 89 | / | 100 | 1.00 | 100 | .81 | 80 | .77 | 85 | .00 | 86 |
| How many days a week do you usually walk to work or to the public transport station? (F6) | .78 | 95 | 1.00 | 100 | .98 | 77 | .62 | 90 | .58 | 85 | .90 | 86 |
| If you walk, how long does it take you to walk to work or to the public transport station? (F7) | .90 | 84 | .93 | 94 | .30 | 62 | .07 | 85 | .17 | 68 | 1.00 | 100 |
| About how many days a week do you usually participate in physical activities/sports in your leisure time? I DO NOT PARTICIPATE IN ANY PHYSICAL ACTIVITIES/SPORTS (F8a) | .83 | 95 | 1.00 | 100 | .42 | 80 | .84 | 96 | 1.00 | 100 | 1.00 | 100 |
| About how many days a week do you usually participate in physical activities/sports in your leisure time? WEEKDAYS (F8b) | .91 | 80 | .96 | 73 | .87 | 85 | .68 | 63 | .77 | 84 | .82 | 83 |
| About how many days a week do you usually participate in physical activities/sports in your leisure time? WEEKENDDAYS (F8c) | .72 | 87 | .94 | 93 | .58 | 57 | .91 | 90 | .85 | 95 | .90 | 83 |
| About how much time a week do you participate in physical activities/sports in your leisure time? WEEKDAYS (F9a) | .74 | 53 | .99 | 93 | .91 | 86 | .80 | 53 | .96 | 89 | .61 | 83 |
| About how much time a week do you participate in physical activities/sports in your leisure time? WEEKENDDAYS (F9b) | .79 | 73 | .88 | 73 | .40 | 64 | .84 | 78 | 1.00 | 95 | .94 | 83 |
| Physical activity is something that I do without really thinking about (F10) | .10 | 34 | .77 | 72 | .74 | 57 | .23 | 19 | .53 | 48 | .71 | 67 |
| I pay for my child to take part in physical activity/sports (F11) | .56 | 61 | .91 | 90 | .90 | 79 | .87 | 98 | .19 | 72 | .62 | 33 |
| I bring my child to physical activity/sport sessions (F12) | .78 | 53 | .76 | 65 | .95 | 84 | .32 | 58 | .77 | 70 | .75 | 33 |
| I encourage my child to take part in physical activity/sports (F13) | -.18 | 11 | .99 | 95 | .63 | 65 | .11 | 30 | .11 | 55 | .38 | 88 |
| I pay attention that my child does enough physical activity/sports (F14) | .14 | 47 | .00 | 95 | .73 | 55 | .46 | 56 | .09 | 55 | .18 | 67 |
| My child is allowed to skip physical activity/sport sessions whenever (s)he wants (F15) | .00 | 95 | .87 | 75 | .72 | 60 | .58 | 80 | .46 | 85 | .92 | 67 |
| I negotiate with my child on how much physical activity/sports (s)he does (F16) | .12 | 39 | .75 | 63 | .78 | 60 | .71 | 79 | .81 | 65 | 1.00 | 100 |
| How often do you tell your child physical activity/sports are good for him/her? (F17) | -.16 | 39 | .96 | 90 | .75 | 65 | .40 | 55 | .74 | 75 | .88 | 88 |
| If I try to prohibit my child from not taking part in physical activity/sport sessions, (s)he will try to skip it anyway (F18) | -.16 | 56 | .73 | 77 | .46 | 90 | .73 | 81 | .59 | 75 | .87 | 86 |
| If I try to prohibit my child from skipping a physical activity/sport session, I find it difficult to stick to my rule(s) if (s)he starts negotiating (F19) | .04 | 65 | .90 | 83 | 1.00 | 100 | .67 | 82 | 1.00 | 100 | 1.00 | 100 |
| I praise my child if (s(he) takes part in physical activity/sports (F20) | .36 | 41 | .72 | 75 | .81 | 65 | .00 | 42 | .74 | 70 | .72 | 72 |
| I punish my child by not allowing him/her taking part in his/her physical activity/sports (F21) | -.11 | 79 | .89 | 95 | .84 | 90 | .67 | 85 | 1.00 | 100 | .00 | 86 |
| I set a time limit on how much time of physical activity/sports my child can do in order to devote more time to his/her homework or other important things (F22) | .09 | 50 | .74 | 65 | .68 | 65 | .38 | 40 | .17 | 47 | .32 | 43 |
| I do not allow my child to take part in physical activity/sports in his/her free time so (s)he can concentrate on his/her studies (F23) | -.23 | 50 | .72 | 63 | .40 | 80 | .30 | 63 | .00 | 95 | .42 | 43 |
| How often do you/your spouse/partner participate in physical activity/sports together with your child? (F24) | .22 | 53 | .87 | 80 | .13 | 35 | .32 | 42 | .81 | 75 | .93 | 50 |
| I let my child participate in physical activity/sports lessons less than I like, because it is too expensive (F25) | -.14 | 53 | .94 | 80 | .59 | 50 | .60 | 68 | .88 | 95 | .25 | 88 |
| About how many hours a day do you usually watch television (including DVDs and videos) in your free time? WEEKDAYS (G1a) | .82 | 47 | .95 | 85 | .83 | 65 | .86 | 65 | .97 | 85 | .96 | 78 |
| About how many hours a day do you usually watch television (including DVDs and videos) in your free time? WEEKENDDAYS (G1b) | .11 | 37 | .86 | 70 | .88 | 60 | .86 | 65 | .79 | 85 | .87 | 44 |
| About how many hours a day do you usually use your computer for activities like chatting online, internet, emailing or playing games on a computer, games console (e.g.Playstation, Xbox, GameCube) during leisure time? WEEKDAYS (G2a) | .91 | 68 | .81 | 65 | .29 | 75 | .78 | 55 | .14 | 85 | .34 | 67 |
| About how many hours a day do you usually use your computer for activities like chatting online, internet, emailing or playing games on a computer, games console (e.g.Playstation, Xbox, GameCube) during leisure time? WEEKENDDAYS (G2b) | .65 | 74 | .97 | 80 | .11 | 75 | .80 | 50 | .79 | 90 | .79 | 89 |
| About how many hours a day do you usually use your mobile phone for calling, texting, playing games or surfing on the internet during leisure time? WEEKDAYS (G3a) | .04 | 58 | 1.00 | 100 | .65 | 80 | .59 | 75 | .84 | 90 | .37 | 78 |
| About how many hours a day do you usually use your mobile phone for calling, texting, playing games or surfing on the internet during leisure time? WEEKENDDAYS (G3b) | .31 | 74 | 1.00 | 100 | .13 | 60 | .58 | 80 | 1.00 | 100 | .76 | 78 |
| Watching television is something I do without even really thinking about (G4) | .36 | 32 | .77 | 40 | .48 | 50 | -.39 | 30 | .78 | 50 | .55 | 56 |
| In general, how often do you watch television during breakfast? (G5a) | .00 | 79 | .91 | 65 | .63 | 90 | .61 | 70 | .49 | 84 | .59 | 78 |
| In general, how often do you watch television during lunch? (G5b) | -.12 | 74 | .84 | 70 | .08 | 80 | .78 | 75 | .66 | 95 | -.04 | 37 |
| In general, how often do you watch television during dinner? (G5c) | .78 | 79 | .90 | 85 | .66 | 70 | .80 | 70 | .69 | 75 | .43 | 67 |
| TV/video/DVD is available in my child’s room (G6) | 1.00 | 100 | 1.00 | 100 | 1.00 | 100 | .77 | 90 | 1.00 | 100 | .00 | 89 |
| I pay attention to the amount of time my child watches TV/video/DVD (G7) | .29 | 37 | / | / | / | / | / | / | / | / | .71 | 57 |
| If my child asks if (s)he is allowed to watch television, I will allow it (G8) | .37 | 42 | .45 | 50 | .65 | 70 | .51 | 55 | .45 | 60 | .79 | 78 |
| My child is allowed to watch TV/video/DVD whenever (s)he wants (G9) | .47 | 26 | .70 | 55 | .66 | 55 | .31 | 25 | .37 | 40 | .89 | 67 |
| I negotiate with my child how much TV/video/DVD (s)he is allowed to watch (G10) | .02 | 37 | .57 | 63 | .78 | 70 | .56 | 40 | .45 | 80 | .79 | 56 |
| How often do you tell your child that watching TV/video/DVD is not good for him/her? (G11) | .07 | 42 | .54 | 83 | .44 | 35 | .57 | 53 | .70 | 75 | -.14 | 33 |
| How often do you tell your child that watching TV/video/DVD can make him/her fat? (G12) | -.13 | 63 | .93 | 85 | .70 | 60 | .54 | 69 | .88 | 90 | .71 | 56 |
| How often do you tell your child that watching TV/video/DVD is bad for his/her eyesight? (G13) | .19 | 53 | .80 | 75 | .57 | 40 | .81 | 74 | .86 | 80 | .72 | 75 |
| If I would like to watch TV/video/DVD, I would restrain myself because of the presence of my child (G14) | .53 | 56 | .92 | 70 | .49 | 55 | .54 | 50 | .81 | 83 | .69 | 56 |
| If I prohibit my child from watching TV/video/DVD, (s)he tries to do it anyway (G15) | .27 | 58 | .93 | 90 | .60 | 80 | .55 | 68 | .89 | 95 | .67 | 63 |
| If I prohibit my child from watching TV/video/DVD, I find it difficult to stick to my rule(s) if (s)he starts negotiating (G16) | .53 | 58 | .70 | 85 | .67 | 65 | .86 | 77 | .95 | 95 | .73 | 88 |
| I allow my child to watch TV/video/DVD as a reward or to comfort him/her (G17) | .36 | 37 | .88 | 90 | .76 | 75 | .65 | 61 | .84 | 80 | .62 | 88 |
| How often do you/r partner watch TV/video/DVD together with your child? (G18) | -.08 | 37 | .92 | 90 | .89 | 60 | .63 | 81 | .95 | 90 | .92 | 44 |
| What do you think of your child’s weight? (H1) | .81 | 83 | 1.00 | 100 | 1.00 | 100 | .43 | 85 | 1.00 | 100 | .96 | 100 |
| Does your child have a set daily routine for bedtime? (H2) | .00 | 95 | 1.00 | 100 | .00 | 85 | / | 100 | .00 | 95 | 1.00 | 100 |
| How many hours of sleep does your child usually have during the night? WEEKDAYS (H3a) | .85 | 74 | .64 | 65 | .68 | 75 | .78 | 74 | .78 | 95 | .75 | 67 |
| How many hours of sleep does your child usually have during the night? WEEKENDDAYS (H3b) | .74 | 56 | .62 | 60 | .85 | 80 | .67 | 47 | 1.00 | 100 | .89 | 88 |
